# Therapeutic Application of Pluripotent Stem Cells: Challenges and Risks

**DOI:** 10.3389/fmed.2017.00229

**Published:** 2017-12-14

**Authors:** Ulrich Martin

**Affiliations:** ^1^Leibniz Research Laboratories for Biotechnology and Artificial Organs (LEBAO), Department of Cardiothoracic, Transplantation and Vascular Surgery, REBIRTH Cluster of Excellence, German Center for Lung Research, Hannover Medical School, Hannover, Germany

**Keywords:** induced pluripotent stem cells, embryonic stem cells, cell product, cellular therapy, risks, good medical practice, clinical translation

## Abstract

Stem-cell-based therapies are considered to be promising and innovative but complex approaches. Induced pluripotent stem cells (iPSCs) combine the advantages of adult stem cells with the hitherto unique characteristics of embryonic stem cells (ESCs). Major progress has already been achieved with regard to reprogramming technology, but also regarding targeted genome editing and scalable expansion and differentiation of iPSCs and ESCs, in some cases yielding highly enriched preparations of well-defined cell lineages at clinically required dimensions. It is noteworthy, however, that for many applications critical requirements such as the targeted specification into distinct cellular subpopulations and a proper cell maturation remain to be achieved. Moreover, current hurdles such as low survival rates and insufficient functional integration of cellular transplants remain to be overcome. Nevertheless, PSC technologies obviously have come of age and matured to a stage where various clinical applications of PSC-based cellular therapies have been initiated and are conducted.

## Introduction

Modern medical technologies with the countless number of available drugs, new diagnostic procedures, and therapeutic applications including complex surgical interventions greatly contributed to a substantially increased life expectancy not only in industrialized countries. On the other hand, the increasing frequency of chronic and age-related diseases, lifestyle diseases, or diseases caused by environmental pollution have created new challenges for medical research, including a clear need to develop novel regenerative therapies.

Stem-cell-based concepts are widely considered as promising, but recent experimental and clinical studies applying various adult stem cells, for instance, for cardiac repair (1), have yielded for the most part disappointing results, in many cases without evidence of any considerable functional improvement or therapeutic effects. Major limitations of the various adult stem and progenitor cell types that had been applied include the restricted potential for culture expansion and especially differentiation into the desired therapeutic cell lineages. Whereas the application of adult stem and progenitors is promising or has already proven successful in case of tissue types that show considerable endogenous potential for regeneration, such as bone or the hematopoietic system (2), treatment of diseases that affect less regenerative organs, such as the brain and the heart (1), has not shown significant therapeutic effects, or the observed therapeutic benefit was due to paracrine effects. Another limitation for experimental evaluation and clinical application of different adult stem and progenitor cells is the typical inability to achieve efficient targeted genome engineering, for instance, to introduce therapeutic transgenes or to correct disease-causing mutations, followed by subsequent isolation of defined transgenic single cell clones. Instead, introduction of therapeutic or reporter transgenes into adult stem and progenitor cells is usually achieved through viral vectors with their well-known drawbacks, including limited transient transgene expression, insertional mutagenesis, and *in vivo* immunogenicity (3).

The availability of human pluripotent stem cells (PSCs), including embryonic stem cells (ESCs) and induced pluripotent stem cells (iPSCs) (4), with their far-reaching potential for proliferation and differentiation, now offers novel opportunities for basic research, disease modeling and drug discovery (5), as well as for the development of cellular therapies.

Intense research has driven forward dramatic progress in virtually all areas of iPSC technology. This includes highly efficient technologies for the transgene-free reprogramming of easily accessible cell sources, such as blood (6), efficient and safe site-specific genetic engineering of iPSCs (7), and the development of technologies for the expansion and differentiation of iPSCs at clinical scale (8). Importantly, also intense research on safety issues of iPSC-based cell transplants, the most critical aspect for clinical applications, is ongoing (9).

The Nobel Prize in 2012 was awarded to S. Yamanaka in particular because the use of iPSCs in disease modeling was already a reality by this time and their immense usefulness in drug development already became obvious. By contrast, and although one single trial using ESCs for treatment of spinal cord injury had already been initiated (10), broad clinical application of PSC-based cellular therapies were not foreseeable in 2012. Five years later, this has changed. A considerable number of ESC-based trials are currently conducted or are recruiting patients. In Japan, people suffering from macular degeneration became the first patients to be treated with iPSC-derived retinal pigment epithelium (RPE), using both autologous and human leukocyte antigen (HLA)-matched allogeneic cells (11). Worldwide, a series of clinical studies are in preparation or ongoing for applications of therapeutic derivatives of PSCs for treatment of macular degeneration, diabetes, neurological disorders, and heart failure (Table 1).

**Table 1:** Therapeutic application of embryonic stem (ES) cell and induced pluripotent stem (iPS) cell products: clinical trials.

Study title	Registration number	Status	(Estimated) enrollment	(Estimated) study completion date	Country	Comments
**ES cells**						
**Cardiac diseases**						
Transplantation of human embryonic stem cell (ESC)-derived progenitors in severe heart failure (ESCORT)	NCT02057900	Phase I study; first patients treated (12), recruiting further patients	6	Jun 2018	France	
**Ophthalmic diseases**						
Stem cell therapy for outer retinal degenerations	NCT02903576	Phase I/II study, recruiting	18	Jun 2019	Brazil	
Clinical study of subretinal transplantation of human embryo stem cell-derived retinal pigment epitheliums (RPEs) in treatment of macular degeneration diseases	NCT02749734	Phase I study; recruiting	15	Dec 2017	China	
Subretinal transplantation of RPEs in treatment of age-related macular degeneration diseases	NCT02755428	Phase I study; recruiting	10	Dec 2020	China	
Treatment of dry age-related macular degeneration disease with RPE derived from human ESCs	NCT03046407	Early phase I study; recruiting	10	Dec 2020	China	
Effect of stem cell on optic atrophy and retinopathy—a preliminary clinical study	ChiCTR-TNRC-11001491	Observational study; recruiting completed	15	Dec 2012	China	Applied stem cell type not defined in the registry
Preliminary clinical studies of stem cells therapy for retinal degeneration diseases	ChiCTR-OCH-14005060	Observational study; recruiting	40	n.a.	China	Applied stem cell type not defined in the registry
The clinical trial of human ESC-derived epithelial cells transplantation in the treatment of severe ocular surface diseases	ChiCTR-OCB-15005968	Observational study; recruiting	20	Dec 2018	China	
Clinical study of subretinal transplantation of human embryo stem cell-derived RPEs in treatment of macular degeneration diseases	ChiCTR-OCB-15006423	Observational study	10	Dec 2017	China	
Clinical study of subretinal transplantation of clinical human embryonic stem cells-derived retinal pigment epitheliums (hESC-RPEs) in treatment of dry age-related macular degeneration diseases	ChiCTR-OCB-15007054	Observational study; recruiting	10	Jun 2017	China	
Safety and efficacy study of OpRegen for treatment of advanced dry-form age-related macular degeneration	NCT02286089	Phase I/II study, recruiting	15	Sep 2019	Israel/USA	
Safety and tolerability of MA09-hRPE cells in patients with Stargardt’s macular dystrophy (SMD)	NCT01625559	Phase I study; not recruiting (exact status unknown)	3	Jun 2015	Korea	
A Phase I/IIa, open-label, single-center, prospective study to determine the safety and tolerability of sub-retinal transplantation of human ESC-derived retinal pigmented epithelial (MA09-hRPE) cells in patients with advanced dry age-related macular degeneration (AMD)	NCT01674829	Phase I/II study; recruiting status unknown	12	Apr 2016	Korea	
A study of implantation of RPE in subjects with acute wet age-related macular degeneration	NCT01691261	Phase I study; active but not recruiting	10	Mar 2017	UK	
A follow up study to determine the safety and tolerability of sub-retinal transplantation of hESC-RPE cells in patients with SMD	NCT02941991	Observational study; follow-up to 5 years of a Phase I/II study; active, not recruiting	11	Dec 2019	UK	
Safety and tolerability of sub-retinal transplantation of hESC-RPE cells in patients with SMD	NCT01469832	Phase I/II study; completed (13)	12	Sep 2015	USA/UK	
Safety and tolerability of sub-retinal transplantation of hESC-derived RPE (MA09-hRPE) cells in patients with advanced dry age-related macular degeneration (Dry AMD)	NCT01344993	Phase I/II study; completed (13)	13	Aug 2015	USA	
Sub-retinal transplantation of hESC-derived RPE (MA09-hRPE) cells in patients with SMD	NCT01345006	Phase I/II study; completed (13)	13	Aug 2015	USA	
Long term follow up of sub-retinal transplantation of hESC-derived RPE cells in SMD patients	NCT02445612	Observational study; long-term follow-up study to NCT01345006; active, not recruiting	13	Dec 2019	USA	
Long term follow up of sub-retinal transplantation of hESC-derived RPE cells in patients with AMD	NCT02463344	Observational study; follow-up study to NCT01344993; active, not recruiting	11	Dec 2019	USA	
Study of subretinal implantation of human ESC-derived RPE cells in advanced dry AMD	NCT02590692	Phase I/II study; recruiting	20	Sep 2022	USA	
A Safety surveillance study in subjects with macular degenerative disease treated with human ESC-derived retinal pigment epithelial cell therapy	NCT03167203	Phase I/II study; not yet recruiting	37	Dec 2029	USA	
**Neurological disorders**						
Safety and efficacy study of human ESC-derived neural precursor cells in the treatment of Parkinson’s disease	NCT03119636	Phase I/II study; recruiting	50	Dec 2020	China	
Dose escalation study of AST-OPC1 in spinal cord injury	NCT02302157	Phase I/II study; recruiting	35	Dec 2018	USA	
Diabetes						
A safety, tolerability, and efficacy study of VC-01™ combination product in subjects with type I diabetes mellitus	NCT02239354	Phase I/II study; active, not recruiting	65	Jan 2021	USA/Canada	
Three year follow-up safety study in subjects previously implanted with VC-01™	NCT02939118	Observational study; enrolling participants by invitation	20	Nov 2021	USA	
A safety and tolerability study of VC-02™ combination product in subjects with type 1 diabetes mellitus	NCT03162926	Phase I study; recruiting	15	Jun 2018	USA/Canada	
A safety, tolerability, and efficacy study of VC-02™ combination product in subjects with type 1 diabetes mellitus and hypoglycemia unawareness	NCT03163511	Phase I/II study; recruiting	55	Dec 2020	USA	
**iPS CELLS**						
**Blood diseases**						
Thalassemia treatment based on the stem cell technology	NCT03222453	Interventional study; enrolling participants by invitation	2	Dec 2017	China	
**Ophtalmic diseases**						
Pilot safety study of iPSC-based intervention for wet-type AMD		Phase I study; one patient done, recruitment terminated in 2015	n.a.	n.a.	Japan	Study not found in registries (source: http://www.riken-ibri.jp/AMD/english/)
Production of iPSC-derived RPE cells for transplantation in AMD	NCT02464956	Observational study; recruiting status unknown	10	Apr 2016	UK	

*The above table is without any claim of completeness*.

*n.a., not available*.

## General Technological Requirements for PSC-Based Cellular Therapies

Meanwhile, many hurdles and limitations for production of clinically applicable ESC and iPSC derivatives have already been overcome. Transgene-free iPSCs can be efficiently derived from easily accessible cell sources such as blood (14, 15). ESCs and iPSCs can now be expanded in scalable suspension culture (8) and large numbers of human PSCs can be produced in fully controlled bioreactors in defined culture media (16, 17). Moreover, sequential inhibition and activation of molecular differentiation pathways has, for the first time, allowed a targeted, more robust and efficient differentiation of human ESCs and iPSCs (18–20). In the meantime, small molecules instead of recombinant proteins are used in such protocols, which have greatly improved the efficiency of differentiation into specific lineages (21–24). The introduction of small molecules as an activator or inhibitor of molecular key pathways has facilitated the development of scalable protocols that are relatively inexpensive and more robust. For various cell lineages, a bottle neck is still the maturation of the PSC-derived cells of interest. It is always a matter of debate whether it is better to transplant mature cells with full functionality (e.g., hepatocytes) or less mature ones that may still have some plasticity and may lead to better structural and functional integration of the graft (e.g., cardiomyocytes with embryonic or fetal phenotype). In other cases, in particular in organs and tissue with substantial stem cell-based regenerative potential (e.g., lung epithelium), it may even be necessary to apply tissue-specific stem cells derived from PSCs in order to achieve long-term functionality.

Also important, highly efficient protocols for a site-specific (and, thus, relatively safe) introduction of transgenes or correction of disease-specific mutations by homologous recombination have been developed based on engineered nucleases, including Zinc finger- and TALE (transcription activator-like effector)-nucleases as well as the CRISPR/Cas9 (clustered regularly interspaced short palindromic repeats; CRISPR-associated proteins) system (25). Utilizing these tools, it has been possible to develop protocols for footprintless gene editing without the need for antibiotic selection (26). Such techniques are of key importance for preclinical animal studies and future cellular therapies as they allow, for example, the correction of disease-related mutations in autologous cells (7) or the well-defined expression of therapeutic transgenes or reporter genes in safe harbor sites that facilitate monitoring of graft survival, functional integration, and distribution in small and large animals (27). Other applications include the targeted introduction of selection genes to enable enrichment of pure cell lineages prior to transplantation (28) or the killing of cellular grafts in case of tumor formation through inducible expression of suicide genes (29).

Another critical hurdle for future stem cell-based therapies that is still not sufficiently addressed is the mode of cell delivery. For most cellular therapeutics, optimal cell application routes and approaches have not been developed yet. In case of heart repair after myocardial infarction, which is a good example for the technical hurdles that have to be overcome, intracoronary infusion was favored for application of bone marrow cells. In this case, it can be observed that a considerable number of the relatively small bone marrow cells is able to cross the vascular wall and is subsequently detectable in the heart muscle (30), but does not form functional myocardium. In the case of the much larger PSC-derived cardiomyocytes, these cells are injected directly into the myocardium, either surgically or via a catheter (31, 32). One shortcoming of this approach is the extensive loss of injected cells directly after application through the injection channel (33). Moreover, although new well-structured functional myocardium is obviously formed, the already existing scar tissue is not replaced and may limit the graft-based functional improvement or may cause arrhythmias (32). Replacement of the whole infarction scar through a tissue engineered myocardial graft that is produced from PSCs may overcome these limitations. However, the problem of proper vascularization of such large dimensional patches has not been solved yet.

## Risks and Hurdles Associated with PSC-Based Cellular Therapies

Besides the development of robust and scalable differentiation protocols, research on safety issues concerning ESC- and iPSC-based cell transplants, including the appearance of genetic abnormalities (34), is probably the most critical aspect for future clinical application. It is necessary to carefully investigate to what extent mutations are already present in the source cells (35), or if they are generated during reprogramming, or are enriched during subsequent hiPSC expansion, which is mandatory for many therapeutic applications. In addition, standard operation procedures (SOPs) are required to routinely check PSCs and their derivatives for abnormalities prior to clinical use. However, many aspects have to be considered during assessment of PSC-related tumor risks. It may well be that risks associated with genetic aberrations are overestimated. It should be emphasized that the detected large number of genetic variations and aberrations in PSCs is somehow misleading since these cells have been investigated for such abnormalities and polymorphisms in much more detail than any other non-tumor cell type. Remarkably, also somatic tissue contains a multitude of genetic variations which do not cause cancer (36). Moreover, the therapeutic tumor risk caused by an individual mutation in PSCs depends to a high degree on the cell type to be transplanted. For instance, terminally differentiated cardiomyocytes are very unlikely to give rise to tumor cells, even if they contain several cancer-related mutations.

Nevertheless, future preclinical risk assessment will definitely require extensive research on tumor and disease risks associated with therapeutic applications of PSC derivatives. Finally, PSC-derived therapeutic cell products will have to undergo extensive characterization, most likely including laborious and relatively expensive techniques such as karyotyping, array comparative genomic hybridization analyses, and whole exome sequencing.

Of course, there are further risks related to the specific graft and its function, the application procedure, the tissue and organ to be treated, and the individual patient. These include, for instance, life-threatening arrhythmia after transplantation of PSC-derived cardiomyocytes, infection risks caused by the application procedure, excessive inflammation due to donor cell death, and immune reactions against transgenic grafts or graft vs. host reactions in case of hematopoietic transplants. Clearly, all these risks have to be considered during development of a regulatory framework for PSC-based cellular therapies.

## General Regulatory Requirements

For clinical applications of ESC and especially iPSC-derived cellular products, the development of more general and therapy/product-specific regulatory guidelines is just in its infancy. At this point, risks are widely unknown. Available reprogramming, culture, gene editing, differentiation, and application techniques are continually being developed and improved, and assays for risk assessment and potency assays have to be defined in close interaction between regulatory authorities, researchers, manufacturers and clinicians.

Doubtless to say, the requirements for standardization and safety of cellular PSC products are demanding. Remarkably, in contrast to most adult stem/progenitor cell types, animal-component-free defined culture media, and matrix formulations have already been developed and iPSC lines have been produced under such conditions (6). Meanwhile, such media are applied in many laboratories for reprogramming and culture expansion. Also, first good medical practice (GMP)-grade culture media are commercially available and other GMP components (e.g., reprogramming kits) are under development by different manufacturers. Although first GXP-conform protocols have already been developed in individual cases, lots off effort will be required for development of SOPs.

Besides development and determination of defined protocols for cell production, product characterization, and clinical application, risk assessment will be of utmost importance for PSC products in general and for individual cell products. The individual risks of teratoma formation due to contaminated undifferentiated cells have to be assessed and minimized. Also, further research focusing on the genetic stability of PSCs is required, especially as genetic abnormalities in their therapeutic derivatives might harbor the risk of tumor formation. However, it is noteworthy to mention that despite leukemia and other tumor cells, there is no other cell type whose genetic integrity, including the occurrence of chromosomal aberrations and nucleotide polymorphisms, has been explored in so much detail as human PSCs. Based on the existing data and ongoing research in the field, suitable assays for risk assessment and subsequent routine characterization of PSC-based cell products will have to be defined.

## Therapeutic Application of PSC-Based Cell Products: Current Clinical Trials

### PSCs for Treatment of Ophthalmic Diseases

The vast majority of the current clinical trials on transplantation of PSC-based cell products aim at cellular therapy of macular degeneration (Table 1). Because the eye is known as an immune-privileged site, the majority of trials that are currently planned or in process use established allogeneic ESC lines. Interestingly more than 20 ESC-based trials of ophthalmic diseases have been registered in various countries including Brazil, China, Israel, Korea, UK, and USA. Among them, single trials have already been completed, and others are currently active or are recruiting patients.

Following approval by the Japanese authorities, two patients were also enrolled in a clinical trial for transplantation of autologous iPSC-derived retinal cells. This is the first clinical trial that is based on an iPSC-based transplant. The first patient underwent treatment for macular degeneration using a hiPSC-derived retinal cell sheet in September 2014 (37). One year after transplantation, there were no serious complications and no unexpected proliferation or sign of local or systemic malignant disease. The transplanted iPSC-RPE sheet showed no sign of rejection until December 2016. Moreover, the best corrected visual acuity of the treated eye did not improve or worsen (http://www.riken-ibri.jp/AMD/english), and it was concluded that the iPSC-based autologous transplantation was safe and feasible in this patient (38).

The second patient enrolled was not treated because of three copy number variations in one patient’s iPSC line that were not detectable in the patient’s original fibroblasts, but that would probably impact expression of affected and surrounding genes (38). The fact that this decision was made, although the conducted safety assays did not provide any evidence that the iPSC-derived RPE cells can be tumorigenic, demonstrates that the iPSC field is well aware of the importance of safety issues. Meanwhile, a second patient has been treated with allogenic iPSC-derived HLA-matched cells (11).

### PSCs for Treatment of Neurological Disorders

The world’s first industry-sponsored clinical trial on a cellular therapy based on human ESCs was initiated in 2010 by Geron, Menlo Park, California, to explore whether hESCs can help nerves to regrow in cases of spinal-cord injury. In 2011, Geron abruptly stopped its stem-cell-therapy programs, including this trial, to focus on its anti-cancer portfolio (39). Even though the trial has officially ended, participants will be monitored for 15 years. In 2017, it was reported that in five treated patients the cell transplant was well tolerated without any detectable immune response despite HLA mismatches, even 10 months after termination of the transient low-dose immunosuppression. The trial was designed as an efficacy study with its low cell dose (5–10× below predicted efficacious range) and suboptimal patient population. However, there was no evidence of significant changes in neurological function and no evidence for ascending loss of function from cells or due to cell application procedure (J. Lebkowski, ISSCR Workshop on Clinical Translation, June 13, 2017).

In 2014, a new Californian company, Asterias Biotherapeutics, started to resurrect the above trial. In 2015, the first patient was treated in a “Phase 1/2a Dose-Escalation Clinical Trial of AST-OPC1 for Complete Cervical Spinal Cord Injury.” In 2017, fist preliminary 9-month efficacy data were presented but final data is pending. Despite these clinical trials for treatment of spinal cord injury, one clinical trial in China has been registered focusing on the development of an ESC-based therapy of Parkinson’s Disease.

### PSCs for Treatment of Diabetes

Four industry-sponsored clinical trials have been registered for treatment of diabetes applying ESC-derived beta cells. Developed and produced by the Californian company Viacyte, pancreatic progenitor cells are used as an islet cell replacement therapy by being transplanted in a device designed to allow direct vascularization of the cells, or by being encapsulated in an immunoprotected device (VC-01™).

### PSCs for Clinical Heart Repair

As yet, there is only one registered clinical trial utilizing PSCs for heart repair. The aim of the “ESCORT”-trial was to demonstrate feasibility and safety of the transplantation of hESC-derived progenitors in severe heart failure. It is noteworthy that no ESC-derived cardiomyocytes have been transplanted and that, although reported by the same group for similar non-human primate progenitors (40), it has not been independently confirmed that the transplanted SSEA-1^pos^ progenitors are able to form functional cardiomyocytes. As yet, treatment of only one patient was reported without complications, such as arrhythmias, tumor formation, or immunosuppression-related adverse events within 3 months of follow-up (12).

However, based on recent advances in relevant technologies including scalable and greatly improved cardiac differentiation efficiencies in defined media (41), different enrichment strategies such as fluorescence-activated cell sorting (42, 43) or metabolic enrichment (44) as well as specification of cardiomyocyte subtypes of these cells (45, 46), it can be presumed that first clinical trials on transplantation of PSC-derived cardiomyocytes will be initiated in the upcoming years.

## Perspectives

Cellular therapies based on ESCs and iPSCs are considered as innovative but complex therapeutic concepts. Addressing the elaborate requirements, basic scientists with leading expertise in hPSC technology, experts in clinical process scale up and toxicology testing, veterinarians and clinician scientists experienced in preclinical large animal models, imaging, and clinical translation of innovative therapies, and experts in ethical and legal aspects of the life sciences have to team up for the development of therapeutic applications and cellular products. Clinical trials that involve standard operating procedures for the production of cellular grafts, quality control, and the application procedure have to be developed together with the competent national authorities, such as the FDA (U.S. Food and Drug Administration) or the EMA (European Medicines Agency).

In most cases, biotech companies with experience in GMP-compliant cell processing and production should also be involved to enable successful clinical implementation and commercial translation.

To date, however, there is not much practical experience in the development of hPSC-based therapeutics, and basic regulatory requirements for adult stem cell therapeutics will need to be adapted. This uncertainty leads to a high capital requirement for possible development costs reducing the calculated return on investment. Therefore, most endeavors will at a first stage focus on non-routine products for individual patients under the umbrella of the so-called “hospital exemption.”

At this stage, it is uncertain when broad clinical application of PSC products can become reality, and whether these applications will be based on patient-specific autologous cells. The production of autologous iPSC-derived cellular therapeutics is generally feasible, but is associated with high costs and would require at least several months for iPSC generation, potential gene editing, differentiation, and quality control.

An alternative approach may be the application of HLA-matched allogeneic iPSC lines from public biobanks, in particular from HLA-homozygous donors, which appears feasible especially in the island nation Japan with its restricted HLA repertoire.

Other approaches enabling the use of allogeneic cells include the induction of peripheral immunological tolerance toward the cell transplant, or the development of PSC lines with substantially reduced allogeneic immunogenicity (47), in particular by targeted genome engineering of the cells HLA-system. However, such approaches are not mature yet, and it remains to be demonstrated whether allotransplantation of such cells will result in long-term engraftment with very limited pharmacological immunosuppression.

## Author Contributions

The author confirms being the sole contributor of this work and approved it for publication.

## Conflict of Interest Statement

The author declares that the research was conducted in the absence of any commercial or financial relationships that could be construed as a potential conflict of interest. The reviewer PH and handling Editor declared their shared affiliation.

## Abbreviations

(h)iPSCs, (human) induced pluripotent stem cells; hPSCs, (human) pluripotent stem cells; ESCs, embryonic stem cells; RPE, retinal pigment epithelium; HLA, human leukocyte antigen.

## References

[1] Jiang M He B Zhang Q Ge H Zang MH Han ZH Randomized controlled trials on the therapeutic effects of adult progenitor cells for myocardial infarction: meta-analysis. Expert Opin Biol Ther (2010) 10(5):667–80. 10.1517/14712591003716437 20384520

[2] Goldstein G Toren A Nagler A. Transplantation and other uses of human umbilical cord blood and stem cells. Curr Pharm Des (2007) 13(13):1363–73. 10.2174/138161207780618759 17506721

[3] Rothe M Modlich U Schambach A. Biosafety challenges for use of lentiviral vectors in gene therapy. Curr Gene Ther (2013) 13(6):453–68. 10.2174/15665232113136660006 24195603

[4] Takahashi K Tanabe K Ohnuki M Narita M Ichisaka T Tomoda K Induction of pluripotent stem cells from adult human fibroblasts by defined factors. Cell (2007) 131(5):861–72. 10.1016/j.cell.2007.11.019 18035408

[5] Shi Y Inoue H Wu JC Yamanaka S. Induced pluripotent stem cell technology: a decade of progress. Nat Rev Drug Discov (2017) 16(2):115–30. 10.1038/nrd.2016.245 27980341 PMC6416143

[6] Haase A Göhring G Martin U. Generation of non-transgenic iPS cells from human cord blood CD34+ cells under animal component-free conditions. Stem Cell Res (2017) 21:71–3. 10.1016/j.scr.2017.03.022 28677540

[7] Merkert S Bednarski C Göhring G Cathomen T Martin U Generation of a gene-corrected isogenic control iPSC line from cystic fibrosis-patient specific iPSCs homozygous for p.Phe508del mutation mediated by TALENs and ssODN. Stem Cell Res (2017) 23:95–7. 10.1016/j.scr.2017.07.010 28925369

[8] Zweigerdt R Olmer R Singh H Haverich A Martin U Scalable expansion of human pluripotent stem cells in suspension culture. Nat Protoc (2011) 6(5):689–700. 10.1038/nprot.2011.318 21527925

[9] Weissbein U Benvenisty N Ben-David U. Quality control: genome maintenance in pluripotent stem cells. J Cell Biol (2014) 204(2):153–63. 10.1083/jcb.201310135 24446481 PMC3897183

[10] Alper J Geron gets green light for human trial of ES cell-derived product. Nat Biotechnol (2009) 27(3):213–4. 10.1038/nbt0309-213a 19270655

[11] Cyranoski D Japanese man is first to receive ‘reprogrammed’ stem cells from another person. Nature (2017). 10.1038/nature.2017.21730 32868921

[12] Menasche P Vanneaux V Hagege A Bel A Cholley B Cacciapuoti I Human embryonic stem cell-derived cardiac progenitors for severe heart failure treatment: first clinical case report. Eur Heart J (2015) 36(30):2011–7. 10.1093/eurheartj/ehv189 25990469

[13] Schwartz SD Regillo CD Lam BL Eliott D Rosenfeld PJ Gregori NZ Human embryonic stem cell-derived retinal pigment epithelium in patients with age-related macular degeneration and Stargardt’s macular dystrophy: follow-up of two open-label phase 1/2 studies. Lancet (2015) 385(9967):509–16. 10.1016/S0140-6736(14)61376-3 25458728

[14] Haase A Olmer R Schwanke K Wunderlich S Merkert S Hess C Generation of induced pluripotent stem cells from human cord blood. Cell Stem Cell (2009) 5(4):434–41. 10.1016/j.stem.2009.08.021 19796623

[15] Schlaeger TM Daheron L Brickler TR Entwisle S Chan K Cianci A A comparison of non-integrating reprogramming methods. Nat Biotechnol (2015) 33(1):58–63. 10.1038/nbt.3070 25437882 PMC4329913

[16] Olmer R Lange A Selzer S Kasper C Haverich A Martin U Suspension culture of human pluripotent stem cells in controlled, stirred bioreactors. Tissue Eng Part C Methods (2012) 18(10):772–84. 10.1089/ten.TEC.2011.0717 22519745 PMC3460618

[17] Kropp C Kempf H Halloin C Robles-Diaz D Franke A Scheper T Impact of feeding strategies on the scalable expansion of human pluripotent stem cells in single-use stirred tank bioreactors. Stem Cells Transl Med (2016) 5(10):1289–301. 10.5966/sctm.2015-0253 27369897 PMC5031176

[18] Burridge PW Thompson S Millrod MA Weinberg S Yuan X Peters A A universal system for highly efficient cardiac differentiation of human induced pluripotent stem cells that eliminates interline variability. PLoS One (2011) 6(4):e18293. 10.1371/journal.pone.0018293 21494607 PMC3072973

[19] Saxena P Heng BC Bai P Folcher M Zulewski H Fussenegger M. A programmable synthetic lineage-control network that differentiates human IPSCs into glucose-sensitive insulin-secreting beta-like cells. Nat Commun (2016) 7:11247. 10.1038/ncomms11247 27063289 PMC4831023

[20] Tao Y Zhang SC. Neural subtype specification from human pluripotent stem cells. Cell Stem Cell (2016) 19(5):573–86. 10.1016/j.stem.2016.10.015 27814479 PMC5127287

[21] Lian X Zhang J Azarin SM Zhu K Hazeltine LB Bao X Directed cardiomyocyte differentiation from human pluripotent stem cells by modulating Wnt/beta-catenin signaling under fully defined conditions. Nat Protoc (2013) 8(1):162–75. 10.1038/nprot.2012.150 23257984 PMC3612968

[22] Huang SX Green MD de Carvalho AT Mumau M Chen YW D’Souza SL The in vitro generation of lung and airway progenitor cells from human pluripotent stem cells. Nat Protoc (2015) 10(3):413–25. 10.1038/nprot.2015.023 25654758 PMC4654940

[23] Kempf H Olmer R Haase A Franke A Bolesani E Schwanke K Bulk cell density and Wnt/TGFbeta signalling regulate mesendodermal patterning of human pluripotent stem cells. Nat Commun (2016) 7:13602. 10.1038/ncomms13602 27934856 PMC5155150

[24] Qi Y Zhang XJ Renier N Wu Z Atkin T Sun Z Combined small-molecule inhibition accelerates the derivation of functional cortical neurons from human pluripotent stem cells. Nat Biotechnol (2017) 35(2):154–63. 10.1038/nbt.3777 28112759 PMC5516899

[25] Gaj T Gersbach CA Barbas CF III ZFN, TALEN, and CRISPR/Cas-based methods for genome engineering. Trends Biotechnol (2013) 31(7):397–405. 10.1016/j.tibtech.2013.04.004 23664777 PMC3694601

[26] Merkert S Wunderlich S Bednarski C Beier J Haase A Dreyer AK Efficient designer nuclease-based homologous recombination enables direct PCR screening for footprintless targeted human pluripotent stem cells. Stem Cell Reports (2014) 2(1):107–18. 10.1016/j.stemcr.2013.12.003 24678453 PMC3966116

[27] Templin C Zweigerdt R Schwanke K Olmer R Ghadri JR Emmert MY Transplantation and tracking of human-induced pluripotent stem cells in a pig model of myocardial infarction: assessment of cell survival, engraftment, and distribution by hybrid single photon emission computed tomography/computed tomography of sodium iodide symporter transgene expression. Circulation (2012) 126(4):430–9. 10.1161/CIRCULATIONAHA.111.087684 22767659

[28] Klug MG Soonpaa MH Koh GY Field LJ. Genetically selected cardiomyocytes from differentiating embronic stem cells form stable intracardiac grafts. J Clin Invest (1996) 98(1):216–24. 10.1172/JCI118769 8690796 PMC507419

[29] Di Stasi A Tey SK Dotti G Fujita Y Kennedy-Nasser A Martinez C Inducible apoptosis as a safety switch for adoptive cell therapy. N Engl J Med (2011) 365(18):1673–83. 10.1056/NEJMoa1106152 22047558 PMC3236370

[30] Valina C Pinkernell K Song YH Bai X Sadat S Campeau RJ Intracoronary administration of autologous adipose tissue-derived stem cells improves left ventricular function, perfusion, and remodelling after acute myocardial infarction. Eur Heart J (2007) 28(21):2667–77. 10.1093/eurheartj/ehm426 17933755

[31] Shiba Y Fernandes S Zhu WZ Filice D Muskheli V Kim J Human ES-cell-derived cardiomyocytes electrically couple and suppress arrhythmias in injured hearts. Nature (2012) 489(7415):322–5. 10.1038/nature11317 22864415 PMC3443324

[32] Chong JJ Yang X Don CW Minami E Liu YW Weyers JJ Human embryonic-stem-cell-derived cardiomyocytes regenerate non-human primate hearts. Nature (2014) 510(7504):273–7. 10.1038/nature13233 24776797 PMC4154594

[33] Martens A Rojas SV Baraki H Rathert C Schecker N Zweigerdt R Substantial early loss of induced pluripotent stem cells following transplantation in myocardial infarction. Artif Organs (2014) 38(11):978–84. 10.1111/aor.12268 24571740

[34] Ronen D Benvenisty N. Genomic stability in reprogramming. Curr Opin Genet Dev (2012) 22(5):444–9. 10.1016/j.gde.2012.09.003 23040504

[35] Abyzov A Mariani J Palejev D Zhang Y Haney MS Tomasini L Somatic copy number mosaicism in human skin revealed by induced pluripotent stem cells. Nature (2012) 492(7429):438–42. 10.1038/nature11629 23160490 PMC3532053

[36] Tomasetti C Li L Vogelstein B. Stem cell divisions, somatic mutations, cancer etiology, and cancer prevention. Science (2017) 355(6331):1330–4. 10.1126/science.aaf9011 28336671 PMC5852673

[37] Reardon S Cyranoski D Japan stem-cell trial stirs envy. Nature (2014) 513(7518):287–8. 10.1038/513287a 25230622

[38] Mandai M Watanabe A Kurimoto Y Hirami Y Morinaga C Daimon T Autologous induced stem-cell-derived retinal cells for macular degeneration. N Engl J Med (2017) 376(11):1038–46. 10.1056/NEJMoa1608368 28296613

[39] Baker M Stem-cell pioneer bows out. Nature (2011) 479(7374):459 10.1038/479459a 22113671

[40] Blin G Nury D Stefanovic S Neri T Guillevic O Brinon B A purified population of multipotent cardiovascular progenitors derived from primate pluripotent stem cells engrafts in postmyocardial infarcted nonhuman primates. J Clin Invest (2010) 120(4):1125–39. 10.1172/JCI40120 20335662 PMC2846046

[41] Kempf H Olmer R Kropp C Ruckert M Jara-Avaca M Robles-Diaz D Controlling expansion and cardiomyogenic differentiation of human pluripotent stem cells in scalable suspension culture. Stem Cell Reports (2014) 3(6):1132–46. 10.1016/j.stemcr.2014.09.017 25454631 PMC4264033

[42] Hattori F Chen H Yamashita H Tohyama S Satoh YS Yuasa S Nongenetic method for purifying stem cell-derived cardiomyocytes. Nat Methods (2010) 7(1):61–6. 10.1038/nmeth.1403 19946277

[43] Dubois NC Craft AM Sharma P Elliott DA Stanley EG Elefanty AG SIRPA is a specific cell-surface marker for isolating cardiomyocytes derived from human pluripotent stem cells. Nat Biotechnol (2011) 29(11):1011–8. 10.1038/nbt.2005 22020386 PMC4949030

[44] Tohyama S Hattori F Sano M Hishiki T Nagahata Y Matsuura T Distinct metabolic flow enables large-scale purification of mouse and human pluripotent stem cell-derived cardiomyocytes. Cell Stem Cell (2013) 12(1):127–37. 10.1016/j.stem.2012.09.013 23168164

[45] Jung JJ Husse B Rimmbach C Krebs S Stieber J Steinhoff G Programming and isolation of highly pure physiologically and pharmacologically functional sinus-nodal bodies from pluripotent stem cells. Stem Cell Reports (2014) 2(5):592–605. 10.1016/j.stemcr.2014.03.006 24936448 PMC4050488

[46] Lee JH Protze SI Laksman Z Backx PH Keller GM. Human pluripotent stem cell-derived atrial and ventricular cardiomyocytes develop from distinct mesoderm populations. Cell Stem Cell (2017) 21(2):179–94.e4. 10.1016/j.stem.2017.07.003 28777944

[47] Gornalusse GG Hirata RK Funk SE Riolobos L Lopes VS Manske G HLA-E-expressing pluripotent stem cells escape allogeneic responses and lysis by NK cells. Nat Biotechnol (2017) 35(8):765–72. 10.1038/nbt.3860 28504668 PMC5548598

